# Deep Learning in Spinal Endoscopy: U-Net Models for Neural Tissue Detection

**DOI:** 10.3390/bioengineering11111082

**Published:** 2024-10-29

**Authors:** Hyung Rae Lee, Wounsuk Rhee, Sam Yeol Chang, Bong-Soon Chang, Hyoungmin Kim

**Affiliations:** 1 Department of Orthopedic Surgery, Korea University Anam Hospital, Seoul 02841, Republic of Korea; drhrleeos@gmail.com; 2 Ministry of Health and Welfare, Government of the Republic of Korea, Sejong 30113, Republic of Korea; rhee1998@snu.ac.kr; 3 Department of Orthopedic Surgery, Seoul National University College of Medicine, Seoul 03080, Republic of Korea; sam310@seoul.ac.kr (S.Y.C.); bschang@snu.ac.kr (B.-S.C.)

**Keywords:** endoscopic spine surgery, neural tissue, image segmentation, computer vision, deep learning

## Abstract

Biportal endoscopic spine surgery (BESS) is minimally invasive and therefore benefits both surgeons and patients. However, concerning complications include dural tears and neural tissue injuries. In this study, we aimed to develop a deep learning model for neural tissue segmentation to enhance the safety and efficacy of endoscopic spinal surgery. We used frames extracted from videos of 28 endoscopic spine surgeries, comprising 2307 images for training and 635 images for validation. A U-Net-like architecture is employed for neural tissue segmentation. Quantitative assessments include the Dice-Sorensen coefficient, Jaccard index, precision, recall, average precision, and image-processing time. Our findings revealed that the best-performing model achieved a Dice-Sorensen coefficient of 0.824 and a Jaccard index of 0.701. The precision and recall values were 0.810 and 0.839, respectively, with an average precision of 0.890. The model processed images at 43 ms per frame, equating to 23.3 frames per second. Qualitative evaluations indicated the effective identification of neural tissue features. Our U-Net-based model robustly performed neural tissue segmentation, indicating its potential to support spine surgeons, especially those with less experience, and improve surgical outcomes in endoscopic procedures. Therefore, further advancements may enhance the clinical applicability of this technique.

## 1. Introduction

In spinal surgery, biportal endoscopic spine surgery (BESS) is a significant advancement over conventional open surgery owing to its advantages [1,2,3], which include smaller incisions, reduced muscle and bone damage, less postoperative pain, and shorter recovery times [4]. High-quality endoscopic equipment markedly enhances image clarity and provides significant assistance to surgeons during procedures. The increased magnification in modern endoscopy allows for the more detailed visualization of critical structures, further enhancing surgical precision. However, despite these advancements, complications, such as dural tears and neural tissue injuries, persist and pose significant challenges during surgery [5,6]. These complications are particularly common among younger surgeons who have not yet reached the learning curve. Among these, dural tears remain the most common and significant complication of endoscopic spinal surgery [5]. The rate of dural tears is reported to be approximately 2.7% [5]. These tears are often managed during surgery by suturing or sealing with specialized products. However, if unnoticed, patients may experience postoperative headaches, nausea, prolonged bed rest, and increased hospitalization and, in severe cases, may require revision surgery. The steep learning curve associated with these complications further limits their widespread clinical adoption, necessitating more experience and skills from surgeons.

Recently, the incorporation of artificial intelligence (AI) into healthcare has been promising, particularly in medical imaging analysis. Deep learning, a subset of AI, has demonstrated remarkable performance in clinical diagnosis and treatment owing to its self-learning capabilities and the ability to extract key features from large datasets [7,8,9]. Semantic segmentation, which is one of the most actively studied fields in computer vision, classifies each pixel of an image into a predefined class. Architectures such as fully convolutional networks (FCN), DeepLab, and Mask R-CNN have been developed and have shown promising results for image datasets comprising common objects [10,11,12]. Studies have demonstrated the effectiveness of deep learning-based segmentation in various medical imaging domains, such as retinal vessel segmentation, tumor detection [13], and instrument tip recognition in spinal surgery [14]. These studies established the foundation for our approach by illustrating the potential of U-Net and similar models for precise segmentation in challenging imaging scenarios [9,13,15].

Despite these advancements, research on the application of deep learning in spinal endoscopy remains limited. Given the critical need to minimize complications, such as dural tears and neural tissue injuries, it is necessary to develop and implement deep learning algorithms for neural tissue recognition in spinal endoscopy. In particular, U-Net and its variants have been widely adopted in biomedical imaging, particularly for small datasets [16,17]. FCN have laid the foundation for pixel-wise segmentation, whereas DeepLab and Mask R-CNN have shown robust performance in handling complex images and multi-object segmentation [15,17,18,19]. We chose U-Net owing to its effectiveness with small biomedical datasets and its capability to capture fine details, thereby making it suitable for neural tissue segmentation in spinal endoscopy [17,20,21]. 

The use of deep learning in spinal endoscopy is relatively new. Studies such as that of Cho et al. [14] focused on the automatic detection of surgical instrument tips to achieve high precision. However, challenges such as differentiating between neural tissues and surrounding structures have not been addressed. Studies on other biomedical images [16,17] have demonstrated the efficacy of U-Net architectures for segmentation, which motivated our choice of model. This study aimed to explore the feasibility of deep learning for neural tissue recognition during spinal endoscopy. By establishing a foundational understanding of how effectively deep learning can identify neural tissues, we hope to pave the way for advancements in real-time tissue recognition, ultimately enhancing the safety and efficacy of endoscopic spinal surgery. 

## 2. Materials and Methods

### 2.1. Dataset

The patient cohort comprised 28 patients, including 21 with lumbar interlaminar decompressions, 5 with lumbar foraminal decompressions, and 3 with cervical foraminotomies. The procedures involved levels 1–2, with six cases involving 2-level surgeries. This dataset is diverse and encompasses a range of demographic profiles, including sex and age. Frames were extracted from each video at 10 s intervals, resulting in approximately 4000 frames. Among these, 2942 frames contained neural tissues that could be detected at the human level. Segmentation labeling was performed using LabelMe by a spinal surgeon (H.R.L., one of the authors.) with >4 years of experience in spinal endoscopic surgery. The dataset was then divided into training, validation, and test sets, with 2307 images (78%) from 22 patients (79%) and 635 images (22%) from 6 patients (21%). We performed a threefold cross-validation on the training/validation set, with each fold comprising 1538 images (52%) for training and 769 images (26%) for internal validation.

The patient demographics for each set are listed in Table 1. The training/validation and test sets did not have overlapping patients, ensuring appropriate validation and preventing the overestimation of performance measures. This study was approved by the Public Institutional Review Board (IRB) of the National Bioethics Policy Institute through the public e-IRB system. The IRB approval number and approval date for this study is “2024-1010-001” and 16 August 2024. The requirement for informed consent was waived by the IRB because of the retrospective nature of this study.

### 2.2. The U-Net Architecture

**Figure 1:**
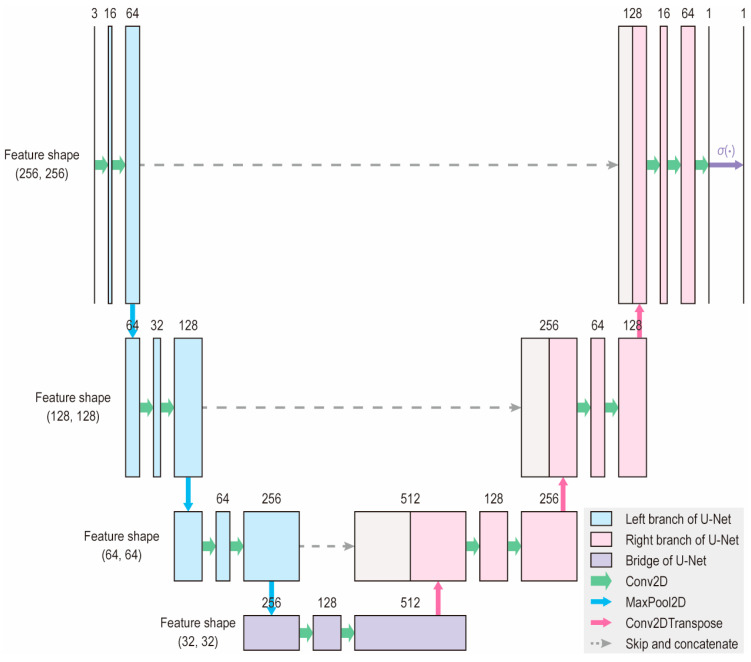
Architecture of the deep learning model. The model accepts an input of shape (256, 256, 3) and produces an output of shape (256, 256, 1). The number of channels is indicated above each block. This architecture is a modified version of the original U-Net, designed to reduce the number of parameters and enable faster learning.

In this study, we trained a deep neural network resembling the U-Net architecture, which has been reported to perform well on the segmentation tasks of small image datasets [16,17]. We selected a U-Net-like architecture based on its demonstrated effectiveness in medical image segmentation, particularly with small datasets [16,17]. U-Net variants can effectively handle limited labeled data, making them suitable for application in spinal endoscopy. As shown in Figure 1, the model had an input shape of (256, 256, and 3) and an output shape of (256, 256, and 1). An input image first undergoes a down-sampling process, also known as the left branch, to extract the features. The bottommost layer or bridge of the network contains the most compressed images with the thickest layers. Subsequently, an upsampling step, or right branch, was performed to recover the original resolution and provide a set of segmentation masks. Skip connections at each level allow for the faster convergence and stability of the deep learning models.

### 2.3. Model Training

**Figure 2:**
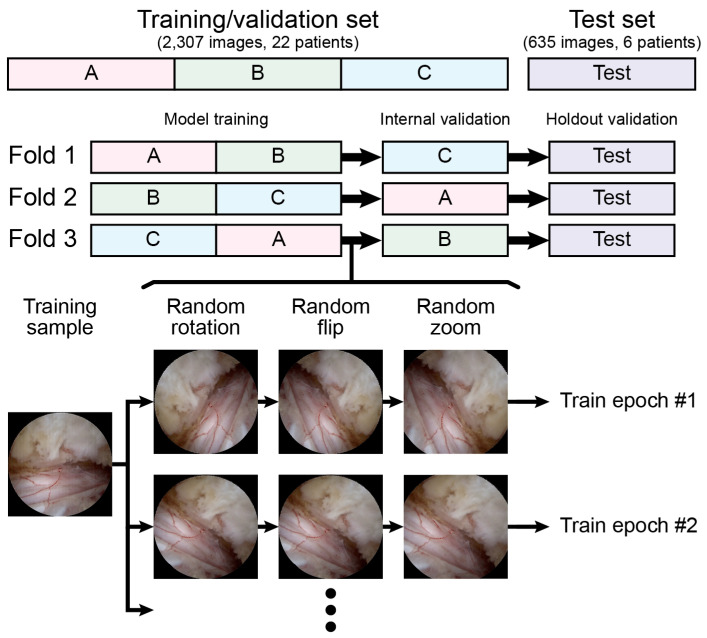
Threefold cross-validation and data augmentation processes with random transform are illustrated.

No preprocessing methods other than resizing or rescaling were applied. We aimed to evaluate the performance of the model under raw conditions to provide more generalized applicability across diverse clinical settings. Data augmentation, which consists of random rotation from −180 degrees to +180 degrees, random flip, and random zoom from 1.0 times to 1.2 times, was applied only during the training of each fold. No augmentation was applied during the internal validation and testing. Unlike common practice, where the training set is expanded by fivefold to tenfold, we did not expand the training set but instead applied random image transforms for each epoch, as illustrated in Figure 2, allowing the model to experience multiple random variations of the original training sample. 

The convolutional layers are randomly initialized with a uniform Glorot distribution, with batch normalization applied before nonlinear activation [22,23]. The full neural network aimed to minimize dice loss, defined as Equation (1), where indices i and j refer to the indices of the rows and columns of pixels, and it was trained for a maximum of 100 epochs (the maximum number of epochs was determined empirically because most trials were terminated before completing 100 epochs owing to early stopping mechanisms) [24]. To prevent overfitting and promote adequate convergence, we incorporated early stopping and learning rate reduction mechanisms. Hyperparameter optimization was performed through a random search of 25 trials, and the search space summarized in Table 2 was determined based on initial experimentation and commonly accepted practices [25].

(1) $$Dice\;Loss=1-\frac{2\times \sum_{i,j}y_{ij}{\hat{y}}_{ij}}{\sum_{i,\;j}y_{ij}+{\hat{y}}_{ij}}$$

After training the models for each fold in the threefold cross-validation process, we generated an ensemble model that averaged the outputs of the models and measured their final performance. All training and testing were performed with TensorFlow 2.14 and Python 3.11, running on a PC with an Intel(R) Core(TM) i9-14900KF CPU, an NVIDIA RTX 4090 24GB graphics card, and 64GB of DDR5 RAM.

### 2.4. Performance Assessment

The performance of the trained model was evaluated using various methods, each of which is described in the following subsections. In this context, a true positive (TP) refers to the intersecting area of the ground truth and predicted masks, a false positive (FP) is defined as the region inside the predicted mask but outside the ground truth mask, and a false negative is the region inside the ground truth mask but outside the predicted mask. In this study, the Dice–Sorensen coefficient (DSC), Jaccard index (IoU), precision, and recall were evaluated for the test set. The image-processing time was also measured to assess the feasibility of the model for analyzing real-time video frames.

#### 2.4.1. Dice–Sorensen Coefficient

The DSC is defined in Equation (2) and is equivalent to the F1-score of a typical two-by-two contingency table. The DSC ranged between 0 and 1, with higher values indicating better performance as the TP increased. Notably, the dice loss is a continuous analog of the negative DSC, and the Dice Loss decreases as the model performance increases.

(2) $$DSC=\frac{2\times TP}{2\times TP+FP+FN}$$

#### 2.4.2. Jaccard Index

The intersection over union (IoU), defined in Equation (3), is the ratio of the intersecting area of the ground truth and prediction masks to their union. Similar to the DSC, its value is always between 0 and 1, and a higher score indicates better performance.

(3) $$IoU=\frac{TP}{TP+FP+FN}$$

#### 2.4.3. Precision and Recall

Precision and recall are defined in Equations (4) and (5) and are widely adopted to measure a model’s performance. Given that their values are dependent on the decision boundaries, the precision–recall curve and the area underneath were also assessed. The area under the precision–recall curve (AUPRC) is also known as average precision (AP).

(4) $$Precision=\frac{TP}{TP+FP}$$

(5) $$Recall=\frac{TP}{TP+FN}$$

#### 2.4.4. Qualitative Assessment

The model performance was qualitatively assessed by exploring the prediction masks obtained from the test set images. This analysis aimed to identify the strengths and weaknesses of our model and discuss strategies for improving its performance in the future.

## 3. Results

### 3.1. Quantitative Results

Table 3 lists the sets of hyperparameters that resulted in the top-10 DSC during the holdout validation with their respective ensemble models. The model that performed the best was trained with a batch size of 12 on an Adam optimizer, with an initial learning rate of 0.00136. The learning rate was reduced by a factor of 0.079 when the loss did not decrease after 7 epochs, and the entire process was terminated when the loss did not decrease after 21 epochs. This set of hyperparameters is in line with commonly accepted practices in medical image segmentation tasks.

**Figure 3:**
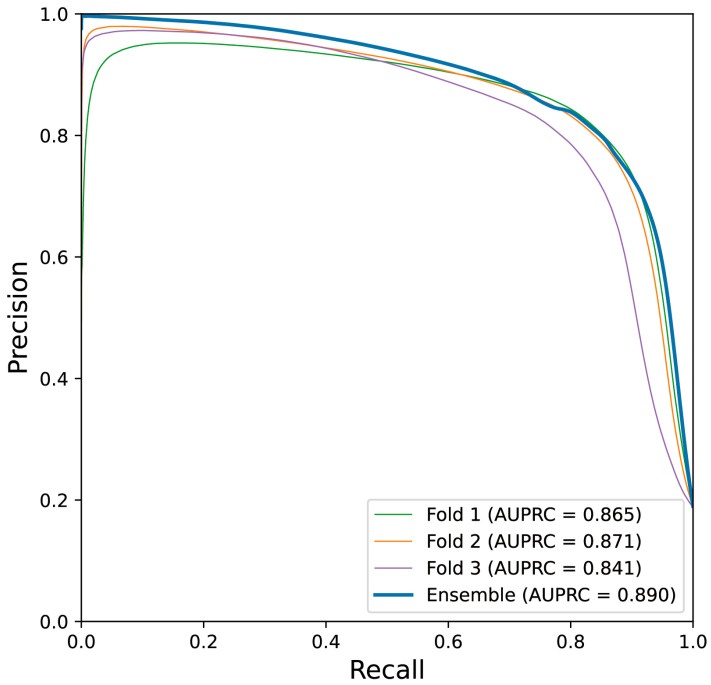
Precision–recall curve obtained from the best-performing trial is shown. Narrow lines indicate the performance of the models trained from each fold on the test set, and the thick line indicates that of the ensemble model.

Table 4 lists the performance measures evaluated from internal and holdout validation, including the performance of all three folds, as well as the ensemble model. The holdout validation performance of each fold did not significantly differ from that of internal validation, and we confirmed that the ensemble model, which is defined by averaging the outputs of models from each fold, generally outperforms each model. The ensemble model exhibited a test DSC and test IoU of 0.824 and 0.701, respectively, and a test precision and test recall of 0.810 and 0.839, respectively. Figure 3 depicts the plotting of the precision–recall curve and AUPRC of the ensemble model and models obtained from each fold.

### 3.2. Qualitative Results

#### 3.2.1. Well-Performing Samples

**Figure 4:**
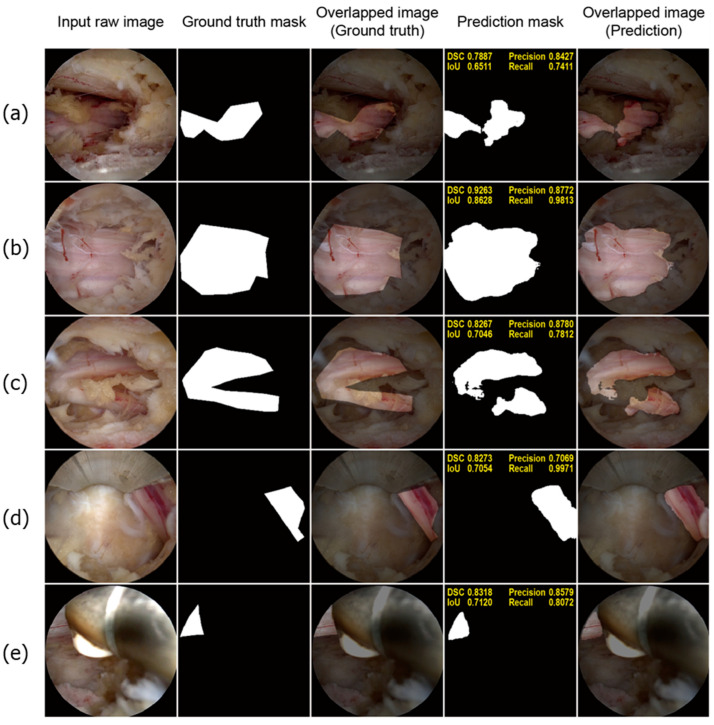
Well-performing test samples. For each sample, the input raw image, ground truth mask, overlapped image of the ground truth mask, prediction mask, and overlapped image of the prediction mask are shown from left to right (**a**–**c**). The deep learning model demonstrated superior accuracy and precision compared to manual annotations. The boundaries predicted by the model are significantly smoother and more precise. Notably, the model accurately identifies epidural fat tissue as non-neural tissue, a distinction that manual annotations often fail to make. This accuracy is evident in the predicted masks (**d**,**e**). Despite the neural tissue being only partially visible in the endoscopic images, the deep learning model accurately detects and represents these small segments of neural tissue. This highlights the model’s impressive performance in recognizing neural tissue in challenging scenarios.

Figure 4 shows a few well-performing test samples. In contrast to the rigid and relatively crude polygon-shaped ground truth masks generated manually using LabelMe, the prediction masks tended to be smoother and more descriptive. Moreover, Figure 4a–c show that the deep learning model effectively learned to exclude perineural fat, which often overlaps with neural tissue. Additionally, the model performed well on challenging samples, where only a small portion of the neural tissue was observed, as depicted in Figure 4d,e. Therefore, it can be concluded that the model successfully learned the distinct features of the neural tissues during spinal endoscopic surgery.

#### 3.2.2. Poorly Performing Samples

**Figure 5:**
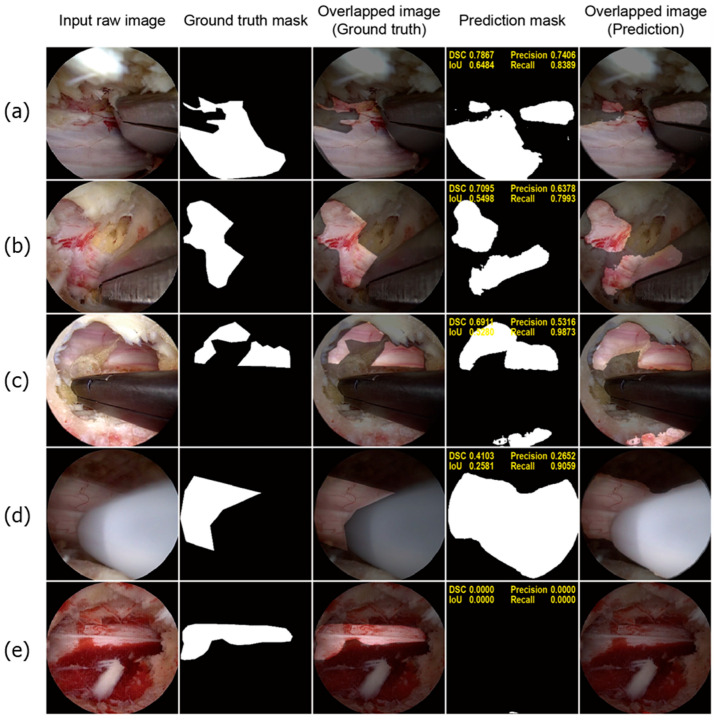
Poorly performing test samples. For each sample, the sequence of images is presented from left to right: the input raw image, ground truth mask, overlapped image of the ground truth mask, prediction mask, and overlapped image of the prediction mask (**a**,**b**). The model misclassified metallic surgical instruments as neural tissue owing to reflections of neural tissue on the metal surfaces (**c**). The model incorrectly identified bloody cancellous bone as neural tissue (**d**). A white instrument used for ablation was also misclassified as neural tissue (**e**). In a highly bloody surgical field, the model failed to detect neural tissue.

To illustrate the limitations of the deep learning model, we present a few poorly performing samples in Figure 5. As shown in Figure 5a,b, the model mistakenly classified some parts of the surgical instruments as neural tissue. Interestingly, the FP regions often correspond to reflections of the neural tissue, suggesting that the model struggled to distinguish between the true neural tissue and its reflections, as both display similar positive features. Similar issues were observed in Figure 5c, where areas with similar morphology and texture to the neural tissue were incorrectly identified. Additionally, as shown in Figure 5d, certain surgical instruments with smooth and tubular shapes, which are typical characteristics of neural tissue, were also misidentified by the model. Furthermore, the model exhibited reduced effectiveness in scenarios with excessive bleeding, as shown in Figure 5e, which affected its overall accuracy in these exceptional cases.

## 4. Discussion

### 4.1. Comparison with Related Studies

Our study highlights the potential of U-Net-based deep learning models for segmenting neural tissues in endoscopic spinal images. With a test DSC of 0.824 and IoU of 0.701, our model demonstrated competitive performance, particularly given the complexity of neural tissue recognition. In comparison to the previous study by Bu et al. [26], which employed Mask R-CNN for tissue segmentation, our U-Net-based approach achieved a higher DSC, which indicates superior neural tissue detection capabilities in endoscopic images. Despite the more challenging context of neural tissue segmentation, the higher AP in our model underscores the efficacy of our methodology. In contrast to the aforementioned study, our deep learning model identified neural tissue, which was more challenging owing to overlapping features with other tissues; therefore, we consider our results notable. Additionally, in clinical settings, accurately distinguishing neural tissue from other soft tissues, such as ligaments and fat, is crucial because they can be confused during surgery. Given the complexity of this task and the clinical necessity of accurately differentiating neural tissue from the surrounding tissues, our study is highly significant as a pilot study, highlighting the feasibility and importance of neural tissue recognition for improving surgical precision and patient outcomes.

Another study utilized Solov2 and Mask R-CNN for tissue and instrument segmentation in spinal endoscopic images, with the best mean AP of 0.735 at approximately 28 frames per second. Our model achieved a better AP with a comparable computational burden, although it may not be a fair comparison considering the differences in task objectives and image resolution. While the 23.2 frames per second achieved by our model may not be sufficient for real-time videos at 30 fps, it could be effective for videos with low sampling rates, such as 15 or 20 fps. Therefore, we conclude that the proposed model can robustly segment neural tissues in real time. Implementing such technology could greatly benefit less-experienced spinal surgeons by providing enhanced guidance during procedures, ultimately serving as an educational tool for junior surgeons.

### 4.2. Clinical Significance

Our qualitative analysis provides valuable insights into the ability of the model to distinguish neural tissues from other tissues, even in scenarios with limited tissue visibility. The deep learning model, trained on manually labeled ground truth masks using LabelMe, demonstrated superior accuracy by correctly excluding fat tissue, which is often mislabeled as neural tissue in manual annotations, as shown in Figure 4a–c. It also performed impressively in recognizing small segments of the neural tissue that are only partially visible in the surgical field. However, the model exhibited limitations, as shown in Figure 5a,b, where metallic surgical instruments were misclassified as neural tissue owing to the reflections of the neural tissue on the metal surfaces. This misclassification, although a shortcoming, indicates that the model has the potential to recognize complex visual patterns, including neural tissue reflections on metals, which extend beyond direct visual cues. These observations highlight the model’s advanced capability in feature detection but also underscore the need for further refinement to reduce false positives associated with instrument reflections and similar tubular structures.

The high positive predictive value (PPV) demonstrates its effectiveness in accurately identifying true neural tissues, which is crucial to ensuring that neural structures are not overlooked during surgery. Our results may not be immediately acceptable for direct application in routine clinical settings. However, this study represents an early exploration of applying deep learning to neural tissue detection, a complex task with high variability in endoscopic images. Even the most skilled surgeons experience fatigue or face challenging surgical environments, which can increase the risk of errors. Our model aims to provide an additional layer of support, ultimately serving as a tool to assist surgeons in reducing preventable mistakes. With further development, including improved model performance and real-time implementation, we believe that such AI-based assistance can complement a surgeon’s expertise and contribute significantly to enhancing patient safety. This high PPV is encouraged, as it reduces the risk of surgeons overlooking critical neural tissues. Therefore, despite the need for improvement in reducing false positives, as highlighted by the challenges associated with the negative predictive value (NPV), the model’s current ability to reliably identify neural tissue remains clinically significant. Its existing capabilities suggest that the model is sufficiently robust to be considered for deployment in clinical settings, offering valuable support during surgical procedures.

Neural tissue recognition during surgery is particularly crucial because many surgical complications, such as dural tears or direct neural injuries, often occur when surgeons fail to detect small, partially obscured neural fibers. The ability of our model to recognize these critical but minimally visible neural structures suggests that it has substantial potential to reduce such surgical risks. Surgeons are more likely to make fewer mistakes when neural structures are fully visible and distinct from the surrounding tissues. However, errors are more common when neural structures are only slightly visible or overlap with other tissues. The success of our model in these nuanced detection tasks highlights its significance, suggesting that it can serve as a valuable tool for enhancing surgical accuracy and reducing the likelihood of complications associated with misidentification.

### 4.3. Limitations and Future Work

A notable limitation of our model was the low NPV, despite the high PPV. We hypothesized that this issue may be influenced by the loss function used during training. Specifically, the dice loss places a greater emphasis on TP regions and does not account for true negative (TN) areas. As a result, TN pixels may not have been adequately trained, potentially leading to a reduced NPV. Implementing binary cross-entropy loss instead of dice loss could potentially improve the NPV, although this might occur at the expense of decreased PPV. Another drawback was the relatively small dataset, which may have affected the generalizability of the model. The current focus on neural tissues limits their applicability in more complex scenarios. Enlarging the dataset by expanding the cohort is the preferred option. However, improvements can also be achieved through enhanced data preprocessing and augmentation techniques. For instance, histogram equalization methods, such as global histogram equalization and contrast-limited adaptive histogram equalization, emphasize the borders of different tissues more prominently, which can result in the improved learning of important features. Additionally, extensive augmentation techniques, such as CutMix and color jittering, may contribute to improved performance because they aid the model in learning more generalized features [24]. Diversifying label entities not only confined to neural tissue may positively affect the model’s performance, because it would be able to learn complicated spatial and temporal relations among different types of objects, allowing them to “think” more like surgeons, who are also heavily dependent on anatomical clues, to distinguish between different types of tissue.

We present this study as a baseline reference and plan to extensively investigate other architectures in the future. Since the publication of U-Net, many of its variants have emerged and have produced better results in segmentation tasks [17]. Residual U-Nets manipulate skip connections within the network to enhance gradient propagation, and the utilization of recurrent convolutional blocks has been reported to improve the performance [25,27]. R2U-Net incorporates these two concepts to achieve superior results [20]. Another variant, named Attention U-Net, uses an attention mechanism to aid the neural network in learning where to “pay attention,” and this method allows the model to have more explainability, which is a crucial aspect of AI, especially in the clinical setting [21].

In this study, we chose not to include preprocessing steps because our primary objective was to develop a model that could perform robustly under raw surgical conditions, thereby increasing its generalizability across diverse clinical environments. By using raw input data, we aimed to validate the model’s effectiveness without relying on preprocessing, which might introduce biases or dependencies that are difficult to standardize in practice. However, in certain scenarios, preprocessing methods such as image normalization, contrast enhancement, or noise reduction could enhance the model performance, particularly for challenging or inconsistent imaging conditions. Future research could explore the addition of preprocessing techniques for specific applications where standardized imaging environments are available, and these methods could help to further improve segmentation accuracy and reduce variability.

## 5. Conclusions

Our study demonstrates the promising potential of U-Net-based deep learning models for neural tissue recognition in spinal endoscopy, achieving a DSC of 0.824 and a Jaccard index of 0.701. These metrics indicate competitive performance compared to similar medical image segmentation tasks. The precision and recall scores of 0.810 and 0.839, respectively, further demonstrate the robustness of our model in accurately identifying neural tissues, even in challenging surgical environments. While the results are encouraging, further research is necessary to enhance the model performance and expand its applicability to diverse tissue types. These advancements could provide significant support to spine surgeons, particularly those with less experience, and ultimately improve the surgical outcomes and patient safety during endoscopic procedures.

## Figures and Tables

**Table 1:** Patient demographics for training/validation and test sets.

	Training/Validation Set	Test Set
Number of images	2307 (78%)	635 (22%)
Number of patients	22 (79%)	6 (21%)
Age (years)	65.4 ± 10.7	63.8 ± 14.1
Sex		
Male	9	3
Female	13	3

**Table 2:** The search space for a random search of hyperparameters is summarized.

Hyperparameter	Search Space
Batch size	{4, 8, 12, 16}
Initial learning rate	loguniform (0.001, 0.1)
Optimizer	{Adam, SGD}
Patience for learning rate reduction	{3, 4, 5, 6, 7}
Reducing factor for learning rate reduction	uniform (0.05, 0.15)

**Table 3:** Sets of hyperparameters that resulted in top-10 DSC in the holdout validation of the ensemble model are shown. Boldfaced numbers indicate the best performance among all trials.

Trial	Hyperparameters					Performance Measures (Holdout Validation)				
	Batch Size	Initial LR	Optimizer	Patience	Reduce Factor	DSC	IoU	Precision	Recall	mAP
1	12	0.00136	Adam	7	0.079	0.824	0.701	0.810	0.839	0.890
2	16	0.00868	Adam	7	0.123	0.817	0.690	0.790	0.845	0.844
3	16	0.00108	Adam	7	0.079	0.815	0.687	0.814	0.815	0.894
4	4	0.01101	SGD	7	0.107	0.814	0.687	0.786	0.845	0.864
5	12	0.00183	Adam	6	0.083	0.809	0.679	0.779	0.842	0.877
6	8	0.09321	SGD	5	0.090	0.809	0.679	0.766	0.857	0.846
7	8	0.03990	SGD	5	0.105	0.803	0.670	0.756	0.856	0.839
8	12	0.00711	Adam	6	0.149	0.798	0.664	0.759	0.842	0.820
9	12	0.01099	Adam	7	0.122	0.797	0.663	0.756	0.845	0.855
10	4	0.00103	Adam	3	0.146	0.794	0.659	0.746	0.849	0.865

**Table 4:** Internal validation and holdout validation results of the trial with the best DSC are shown. Results of models trained from each fold as well as the ensemble model are provided.

Internal Validation	DSC	IoU	Precision	Recall	AUPRC
Fold 1	0.818	0.692	0.808	0.828	0.849
Fold 2	0.815	0.688	0.821	0.809	0.868
Fold 3	0.810	0.680	0.805	0.814	0.870
Holdout validation	DSC	IoU	Precision	Recall	mAP
Fold 1	0.827	0.705	0.813	0.841	0.865
Fold 2	0.820	0.694	0.810	0.829	0.871
Fold 3	0.792	0.656	0.806	0.780	0.841
Ensemble	0.824	0.701	0.810	0.839	0.890

## Data Availability

Data related to the findings of this study are available from the corresponding author upon reasonable request.
